# Estimation of Wave Period from Pitch and Roll of a Lidar Buoy

**DOI:** 10.3390/s21041310

**Published:** 2021-02-12

**Authors:** Andreu Salcedo-Bosch, Francesc Rocadenbosch, Miguel A. Gutiérrez-Antuñano, Jordi Tiana-Alsina

**Affiliations:** 1 CommSensLab-UPC, Department of Signal Theory and Communications, Polytechnic University of Catalonia (UPC), C/ Jordi Girona, 1-3, E-08034 Barcelona, Spain; andreu.salcedo@upc.edu (A.S.-B.); miguel.angel.gutierrez@upc.edu (M.A.G.-A.); 2 Institut d’Estudis Espacials de Catalunya (IEEC), UPC, c/ Gran Capità, 2-4, E-08034 Barcelona, Spain; 3 Nonlinear Dynamics, Nonlinear Optics and Lasers (DONLL), Department of Physics (DFIS), UPC, C/ Rambla Sant Nebridi, s/n, E-08222 Terrassa, Spain; jordi.tiana@upc.edu

**Keywords:** wave, period, tilt, pitch, roll, PSD, Blackman–Tukey, IMU, lidar, buoy

## Abstract

This work proposes a new wave-period estimation (L-dB) method based on the power-spectral-density (PSD) estimation of pitch and roll motional time series of a Doppler wind lidar buoy under the assumption of small angles (±22 deg) and slow yaw drifts (1 min), and the neglection of translational motion. We revisit the buoy’s simplified two-degrees-of-freedom (2-DoF) motional model and formulate the PSD associated with the eigenaxis tilt of the lidar buoy, which was modelled as a complex-number random process. From this, we present the L-dB method, which estimates the wave period as the average wavelength associated to the cutoff frequency span at which the spectral components drop off L decibels from the peak level. In the framework of the IJmuiden campaign (North Sea, 29 March–17 June 2015), the L-dB method is compared in reference to most common oceanographic wave-period estimation methods by using a Triaxys^TM^ buoy. Parametric analysis showed good agreement (correlation coefficient, ρ = 0.86, root-mean-square error (RMSE) = 0.46 s, and mean difference, MD = 0.02 s) between the proposed L-dB method and the oceanographic zero-crossing method when the threshold L was set at 8 dB.

## 1. Introduction

In the last few decades, there has been rising interest in offshore wind energy due to higher and more homogeneous winds that can be found in open sea environments [1]. High investments in offshore-wind-farm deployment and operation have been made in Europe in recent years [2]. However, offshore wind energy (WE) is still one of the most expensive energy sources [3] and needs cost optimization in order to achieve commercial competitiveness. One of the main concerns in the WE industry is obtaining trustworthy data to assess the feasibility of future offshore-wind-farm locations. Meteorological masts (metmasts) have been traditionally used for this purpose. However, their high cost has produced the need for alternative atmosphere-assessment methods.

Floating Doppler wind lidars (DWLs) are one of the most suitable candidates to be accepted in the WE industry [4] as a replacement for costlier metmasts. When placed over platforms or buoys, DWLs can assess wind resources in a cost-effective way [5,6]. Moreover, they can be redeployed at multiple locations, being able to cover large areas and offering high versatility [7]. On the other hand, floating DWLs suffer wave-induced errors on wind measurements [8]. Sea waves induce translational (sway, surge, and heave for the *x*, *y*, and *z* axes, respectively) and rotational (roll, pitch, and yaw for the *x*, *y*, and *z* axes, respectively) motion to the floating DWL, which accounts for 6 degrees of freedom (DoF), creating a Doppler effect over the wind vector retrieval and turbulence intensity (TI) [9,10,11,12,13,14,15], with errors of about 10% in horizontal wind speed (HWS). and 40% in TI [11].

Lidar buoys are usually wave buoys moored to the seabed by means of a clump. The buoy’s design is a trade-off between accurate lidar wind measurements and attitude measurements for wave-induced motion compensation [16]. Of the 6 DoF of a wave buoy (sway, surge, heave, roll, pitch, and yaw), sway, surge, and yaw are mainly determined by wind and current forces, whereas heave, roll, and pitch are mainly determined by sea state [10] and are used to study sea waves [17]. Sea waves are a subject of interest in various fields such as marine engineering [18], oceanography [17,19,20,21], and wind engineering [22,23]. Waves can be studied from directional and nondirectional perspectives by means of the directional and nondirectional spectra, which are estimated from measuring a wave buoy’s heave, roll, and pitch records [17,24]. From spectrum estimations, wave period and height variables are derived and studied.

In this study, we present a spectral-analysis methodology to estimate the wave period from roll and pitch records (2 DoF) measured by a lidar buoy and reconcile our methodology to classical oceanographic wave-period estimation methods in the state-of-the-art, which usually rely on average and zero-crossing period computation. We assumed quasistatic yaw rotation and neglected translational motion on account of the buoy’s mooring topology.

Incipient studies addressed the topic as follows: in [25], the wave-induced buoy’s tilt period was computed from the smoothed fast Fourier transform (FFT) of pitch and roll time series. The most prominent peak of these 2 FFTs was chosen as the most relevant spectral component, and the period was estimated as the inverse of the frequency corresponding to it. In [11], the roll and pitch tilt periods were virtually correlated (ρ≃0.5); thus, 1 DoF was considered informative of the buoy’s motional wave period. In [26], two estimation methods to assess the wave period from pitch and roll measurements based on Blackman–Tukey power-spectral-density (PSD) estimation method were presented. Because the correlation between pitch and roll periods showed up experimentally, estimations using 1 DoF (either roll or pitch) became meaningful for the study. The first, the peak method, estimated the period as the inverse of the frequency of the maximum of the PSD. The second, the 3 dB method, defined the 3 dB threshold as the frequency region containing PSD values higher than half of the PSD maximum. In this method, the period was estimated as the inverse of the average of the start and stop cutoff frequencies of the 3 dB region. The 3 dB method yielded much higher correlation coefficients (ρ = 0.62) than those of the peak method (ρ = 0.37) when compared to the measured wave periods from reference buoys. However, a formulation was missing explaining the 3 dB PSD approach in relation to the different oceanographic definitions existing in the state-of-the-art for the wave period or the underlying foundations.

Here, we present the sought-after formulation of the 3 dB method in relation to well-established wave-period oceanographic definitions. We also extend our pitch and roll spectral analysis (2 DoF) to the derivation of the tilt-angle PSD (so-called buoy eigenangle) representing the combined rotation effects of pitch and roll angles on buoy rotation geometry.

This paper is structured as follows: Section 2 begins with a description of the experimental setup at IJmuiden (North Sea) as part as of the validation trials of the lidar buoy prototype and describes the used methodology. The latter revisits the spectral foundations of the estimation of the sea-wave period (Section 2.2), presents our simplified 2 DoF buoy motion model and related eigenangle PSD formulation (Section 2.3), and presents the so-called L-dB PSD method (the name is derived from the 3 dB method; Section 2.4 and Section 2.5). Section 3 discusses the results and carries out a parametric study in order to quantitatively relate the L-dB threshold, which was used in the spectral context (PSD), to the oceanographic context (wave sensors). Lastly, Section 4 gives concluding remarks.

## 2. Materials and Methods

### 2.1. Materials

**Figure 1:**
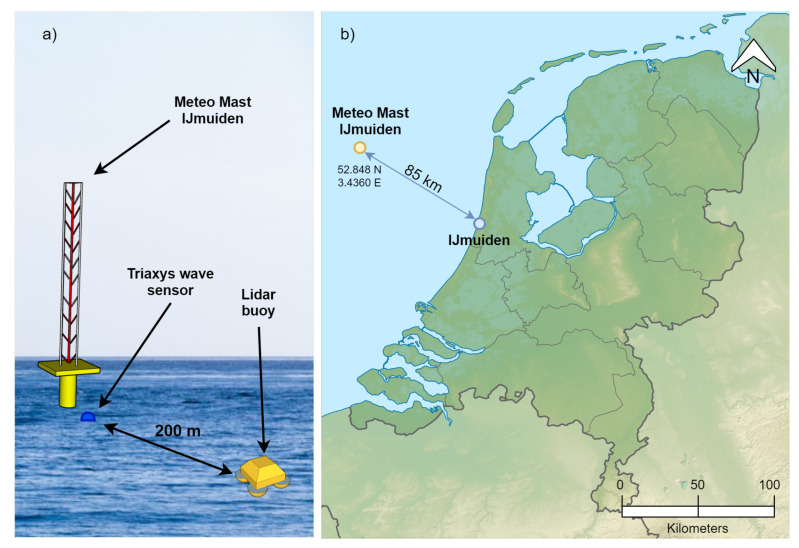
IJmuiden test campaign: (**a**) experiment setup at IJmuiden’s test showing the lidar buoy prototype test Doppler wind lidar (DWL) buoy and reference meteorological mast (metmast), adapted from [26], and (**b**) location map adapted from [30].

In 2015, the validation campaign of the lidar buoy prototype test floating lidar buoy at the IJmuiden test site (North Sea) took place [27]. IJmuiden is a coastal city that hosts the sea lock at the entrance of the North Sea Canal providing access to the Amsterdam (Netherlands) port region. The experimental campaign aimed to assess the wind measurement accuracy of the lidar buoy against the reference meteorological mast (IJmuiden) [28]. Next to the metmast, a Triaxys^TM^ wave buoy measured the main wave and current parameters. The main instruments used in this study were (i) a 3DM-GX3-45 inertial measurement unit (IMU) on the lidar buoy measuring the buoy’s tilt (roll, pitch, and yaw); accelerations in the *x*, *y*, and *z* axes; and global-positioning-system (GPS) position at a sampling rate of approximately 8 Hz and (ii) a Triaxys^TM^ wave buoy next to the metmast measuring the reference wave parameters at a sampling period of 1 h [29]. Figure 1 shows the instrumentation setup of the campaign and the location of IJmuiden’s test facilities. For this study, 1920 wave-buoy data records from 29 March to 17 June (80 days) were used.

**Figure 2:**
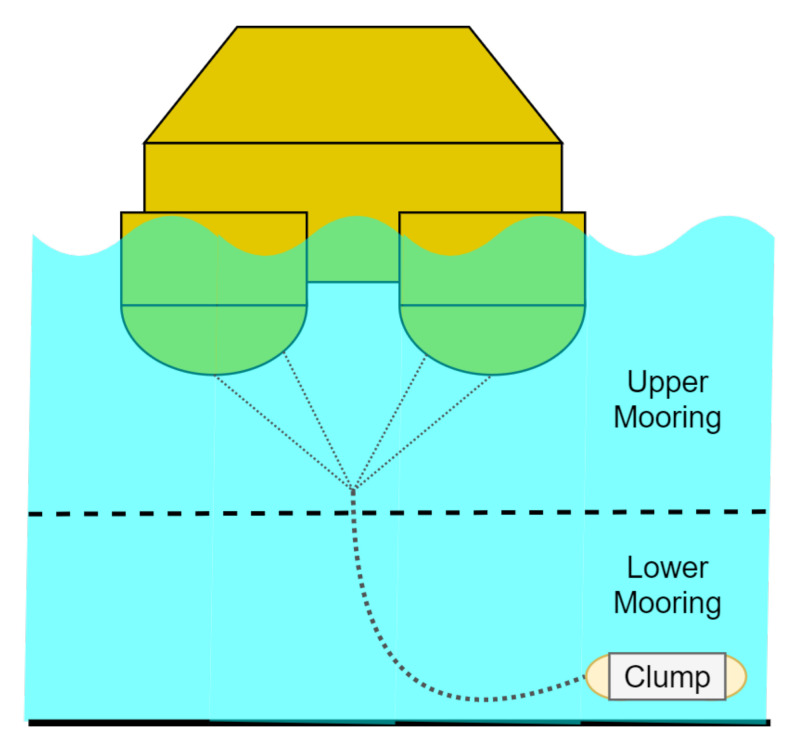
Mooring system scheme of the lidar buoy prototype used in the IJmuiden campaign.

*Lidar buoy prototype*: the test buoy was a precommercial lidar buoy specially optimized to host a ZephIR^TM^300 lidar [27]. It had 3.77 m width, weighed 3 tons, and had a modular four-floater structure designed to satisfy wind-energy measurement requirements and to perform wave measurements from buoy accelerations [16,31]. It was equipped with additional sensors in order to measure a wide variety of wind- and sea-related data. Specifically, it hosted a MicroStrain 3DM-GX3-45 IMU combining a high-precision GPS unit, an accelerometer, and a gyro. The gyro measures Euler’s angles (roll, pitch, and yaw), the accelerometer measures translational accelerations on these axes, and the GPS module measures the position of the buoy. An extended Kalman filter was applied over the IMU measurements in order to track the buoy’s attitude [31]. However, only altitude recorded higher than 0 m were available. From one of the 4 corners of the buoy, a mounted tail acted as a “stern” for the buoy, so that the opposite corner faced the wind direction. The buoy was moored to the seabed by a mooring system consisting of two main parts: (i) upper mooring consisting of four lines connected to each of the buoy’s floaters united in its bottom to a single line and (ii) lower mooring consisting of a clump weight (see Figure 2).

*Triaxys wave buoy*: The Triaxys^TM^ wave buoy is a wave sensor designed for accurate measurement of directional waves and currents at a sampling period of 1 h. It is equipped with 3 accelerometers, 3 gyroscopes, and a compass [32] in order to measure the most relevant directional and nondirectional wave parameters. Some of the parameters yielded by the wave sensor were wave-height definitions ($H_{max}$, $H_{10}$, $H_{sig}$, and $H_{avg}$), wave-period definitions ($T_{max}$, $T_{10}$, $T_{sig}$, $T_{z}$, $T_{avg}$, $T_{p}$, and $T_{p5}$), MeanDirection, and MeanSpread. Subindices max, 10, sig, avg, z, p, and p5 refer to the maximal wave height and its corresponding period ($H_{max}$ and $T_{max}$, respectively), the highest tenth of the waves’ average height and period ($H_{10}$ and $T_{10}$, respectively), the highest third of the waves’ average height and period ($H_{sig}$ and $T_{sig}$, respectively), the average wave height and period ($H_{avg}$ and $T_{avg}$, respectively), the average zero upcrossing period ($T_{z}$), the period corresponding to the highest spectral component of the wave energy spectrum ($T_{p}$), and the peak wave period computed by the READ method ($T_{p5}$), respectively [33]. Wave period parameters $T_{z}$, $T_{avg}$ and $T_{p}$ are formulated in Section 2.2.

Triaxys^TM^ computes these parameters from heave, pitch, and roll measurements estimated from the 6 DoF measurements by 3 accelerometers and 3 gyros by solving the nonlinear differential equations relating the buoy motion to accelerations and angular rates. It follows a similar procedure to that in [34] to obtain heave, surge, and sway translational motions and roll, pitch, and yaw rotational motions. Wave analysis was then carried out on the buoy by performing zero-crossing analysis of wave elevation in the time domain, nondirectional analysis by means of FFT methods, and lastly directional wave analysis [33].

### 2.2. Method (I): Estimation of Sea-Wave Period

Waves can be analysed from directional and nondirectional perspectives depending on the purpose of the study and the available data. Directional wave analysis studies the contribution of ocean waves propagating in different directions with different amplitudes and periods by means of the directional spectrum (DS(f,θ)) of the wave heave by means of the two slope components of the buoy, computed from roll and pitch records [35,36], with techniques such as the Fourier expansion method (FEM) and the maximum entropy method (MEM) [37]. Nondirectional wave analysis studies surface ocean waves from the nondirectional energy spectrum (S(f), computed from wave elevation) [17,21]. S(f) is defined as [38]

(1) $$S\left(f\right)=FT\left(H\left(t\right)\right)=\int_{0}^{T}H\left(t\right)e^{-i2\pi ft}dt,$$

where H(t) is the wave elevation as a function of time, t, f is frequency, and T is the study period.

Directional and nondirectional wave spectra are related in the following way:

(2) DS(f,θ)=S(f)D(f,θ),

where D(f,θ) is the directional spreading function and θ is the wave angular direction. S(f) in Equation (1) above can also be re-encountered by integrating DS(f,θ) over all angular directions (θ from 0 to 2π), $S\left(f\right)=\int_{0}^{2\pi }D\left(f,\theta \right)d\theta$.

Spectral moments $m_{n}$ are defined as

(3) $$m_{n}=\int_{0}^{\infty }f^{n}S\left(f\right)df,$$

where n stands for an nth order moment.

Different wave amplitude and period characterization parameters can be derived from spectrum S(f) and its spectral moments $m_{n}$. Some of the most relevant wave-period definitions are as follows [35]:

- mean zero-crossing period, which is defined as
  (4) $$T_{z}=\sqrt{\frac{m_{0}}{m_{2}}};$$
- average period
  (5) $$T_{avg}=\frac{m_{0}}{m_{1}};$$
- and peak period
  (6) $$T_{p}=\frac{1}{f_{p}},$$
  where $f_{p}$ is the peak frequency of S(f).

### 2.3. Method (II): Buoy-Motion Model

**Figure 3:**
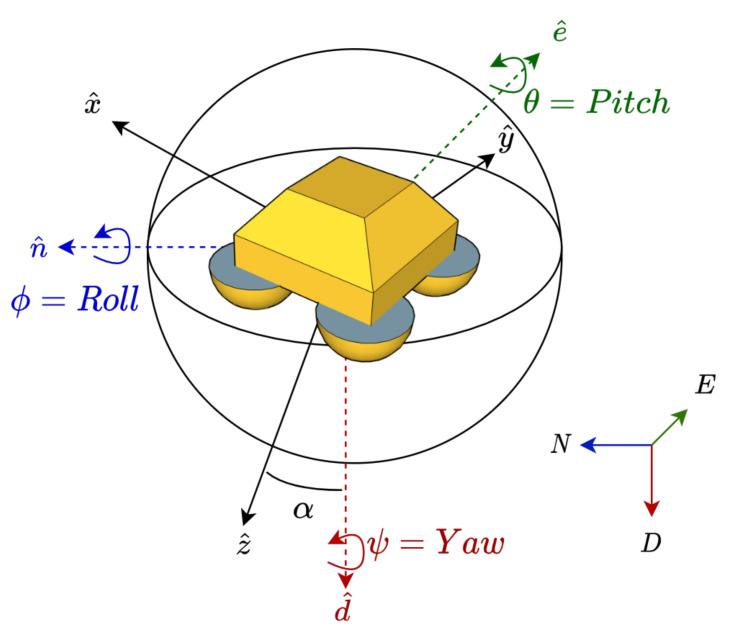
Fixed and moving-body (buoy’s) coordinate systems used: the fixed coordinate system is the right-handed north–east–down (NED) system (dashed arrows with unitary vectors $\hat{n}$, $\hat{e}$, and $\hat{d}$ plotted in blue, green, and red, respectively). The buoy’s coordinate system is denoted as XYZ (solid arrows with unitary vectors $\hat{x}$, $\hat{y}$, $\hat{z}$). α is the buoy’s eigenangle defined as the angle between unitary vectors $\hat{d}$ and $\hat{z}$.

We defined the buoy’s *moving body* Cartesian right-handed XYZ coordinate system and the *global* Cartesian right-handed north–east–down (NED) frame of reference (Figure 3). Without external forces, the *x*, *y*, and *z* axes of the buoy’s *moving body* XYZ coordinate system would be aligned with the north, east, and vertically down axes of the *global* NED frame of reference.

**Figure 4:**
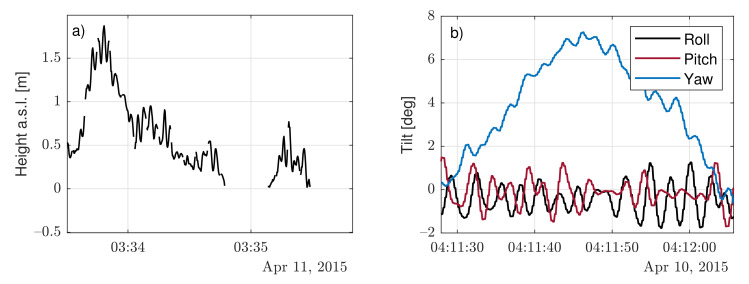
Motional temporal series (Ijmuiden campaign): (**a**) heave signal above sea level (a.s.l.) on 11 April 2015 and (**b**) roll, pitch, and yaw signals on 10 April 2015).

In practice, such external forces can cause translational motion in the N, E, and D directions (surge, sway, and heave, respectively), and rotational motion along the N, E, and D axes (roll, pitch, and yaw, respectively) to the buoy [9,14]. The buoy’s mooring limits surge and sway motion, while heave (Figure 4a) follows the wave altitude. Regarding rotational motion, *roll* and *pitch* are mainly characterized by wave-motion behaviour, whereas *yaw* motion is mainly determined by the wind and currents due to the buoy’s tail acting as the stern. Therefore, heave (translational), and the roll and pitch (rotational) motions are the most informative parameters of wave motion.

The yaw motion showed slow variations with time (typically greater than 1 min; quasistatic approximation) due to wind and current influence, whereas the roll and pitch motions exhibited comparatively much faster oscillatory behavior due to wave influence (see Figure 4b) on time scales of the order of seconds.

The IMU was set up to measure the buoy’s rotation angles on the basis of the fixed global right-handed NED coordinate system (see Figure 3). We defined $\hat{n}$, $\hat{e}$, and $\hat{d}$ as the unitary vectors aligned with the N, E, and D axes, respectively. On the other hand, we defined the $\hat{x}$, $\hat{y}$ and $\hat{z}$ unitary vectors along the rotated moving-body coordinate system (XYZ).

*Large-angle case*: in order to express our rotation problem with a single angle (so-called eigenvector-axis-associated angle or eigenangle for short in what follows, denoted α in Figure 3) we resorted to Euler’s rotation theorem, which states that every rotation in three dimensions is defined by its axis (a vector along this axis is unchanged by the rotation) and its angle (the amount of rotation about that axis). Euler’s theorem also states that any 3D body rotation can be described by three angles. Therefore, the eigenangle can be expressed from the roll, pitch, and yaw rotation angles. There are many different mathematical conventions for these three angles depending on the axes where the rotations are carried out and its order. We used the D-E-N convention defining the specific sequences of axes rotation (D-E-N axes are the global-coordinate (GPS) axes or fixed counterparts of Z-Y-X axes attached to a moving body, i.e., the buoy). In the D-E-N convention, we have three composed elemental rotations carried out sequentially in the global fixed coordinate axes: first, around the D axis (yaw motion, denoted by ψ); second, around the E axis (pitch, θ); and third, around the N axis (roll, ϕ), see Figure 3. The angles were positive counterclockwise. Considering these definitions, Euler’s rotation matrix can be formatted as follows [39]:

(7) $$R=R_{\psi }R_{\theta }R_{\phi },$$

where $R_{\phi }$, $R_{\theta }$, and $R_{\psi }$ are the counterclockwise extrinsic rotation matrices around the N axis (roll), E axis (pitch), and D axis (yaw), respectively.

(8) $$\begin{array}{cc} R_{\phi } & =\left(\begin{array}{ccc} 1 & 0 & 0\\ 0 & \cos\phi & \sin\phi \\ 0 & -\sin\phi & \cos\phi \end{array}\right),\\ R_{\theta } & =\left(\begin{array}{ccc} \cos\theta & 0 & -\sin\theta \\ 0 & 1 & 0\\ \sin\theta & 0 & \cos\theta \end{array}\right),\\ R_{\psi } & =\left(\begin{array}{ccc} \cos\psi & \sin\psi & 0\\ -\sin\psi & \cos\psi & 0\\ 0 & 0 & 1 \end{array}\right). \end{array}$$

Then, any vector rotation can be described by multiplying it with the rotation matrix:

(9) $$\vec{r_{rot}}=R\vec{r},$$

where $\vec{r}$ and $\vec{r_{rot}}$ are vectors (before and after rotation, respectively) with coordinates expressed in the fixed-coordinate system. We define α as the eigenvector-axis-associated angle of the buoy’s combined motion in the roll, pitch, and yaw angles. Conceptually, α is the angle between the down axis of the global coordinate system (NED) and the Z axis of the buoy (moving body, XYZ) (see Figure 3). As previously mentioned, the D and Z axes are described by unitary vectors $\hat{d}=\left[0,0,1\right]$ and $\hat{z}$, respectively. Given $\hat{d}$ (fixed coordinate system), vector $\hat{z}$ (moving coordinate system) can be expressed in the fixed coordinate system using Equation (9):

(10) $$\hat{z}=R\hat{d}=R_{\phi }R_{\theta }R_{\psi }\hat{d}=\left[\begin{array}{c} -\sin\theta \\ \sin\phi \cos\theta \\ \cos\phi \cos\theta \end{array}\right].$$

**Figure 5:**
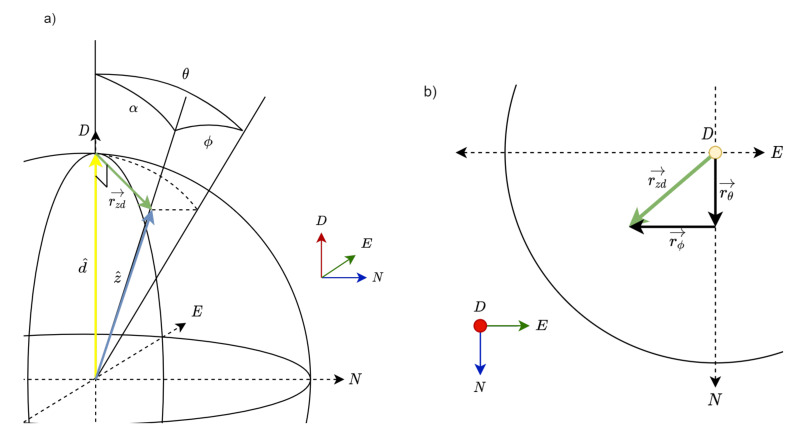
Geometrical representation of a buoy’s rotation in the roll and pitch dimensions of movement and vector approximation for small angles: (**a**) three-dimensional geometry sketch showing eigenangle α, roll (ϕ), and pitch (θ) angles and vectors $\hat{d}$ and $\hat{z}$ in an NED coordinate system. $\hat{d}$ transforms into $\hat{z}$ after the roll (ϕ) and pitch (θ) rotations about the N and E axes, respectively (Equation (12)). (**b**) Representation of roll and pitch rotations $\vec{r_{\phi }}$ and $\vec{r_{\theta }}$, respectively, on the NE plane (Equation (10)) along with the resultant vector $\vec{r_{zd}}$ (Equation (14)).

This can be seen graphically in Figure 5a, where $\hat{z}$ is the result of rotating $\hat{d}$ by θ deg around E (pitch) and ϕ deg around N (roll). Because of the D-E-N convention to describe chained rotations, α was invariant to the yaw rotation around D axis. Similarly, α is invariant to heave, which is translational motion along the D axis.

Then, α can be computed from the dot product between $\hat{d}$ and $\hat{z}$ as follows:

(11) $$\alpha =\arccos\left(\hat{d}\cdot \hat{z}\right).$$

Inserting $\hat{d}=\left[0,0,1\right]$ and $\hat{z}$ (Equation (10)) into Equation (11) yields

(12) α=arccos(cosϕcosθ).

Because the cosine is an even function, α in Equation (12) above is always positively defined.

**Figure 6:**
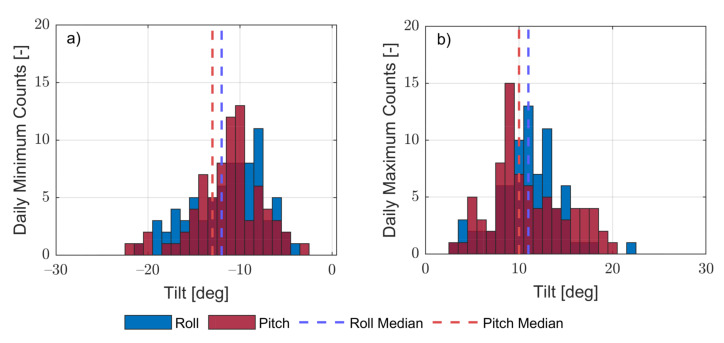
Histograms of the daily minimal and maximal roll and pitch inertial-measurement-unit (IMU) records (57,520,000 records between 29 Match and 17 June): (**a**) daily minimal tilt-record histogram and (**b**) daily maximal tilt-record histogram. The dashed lines represent roll (blue) and pitch (red) medians in both panels.

*Small-angle case*: Figure 6 plots 80 day histograms describing maximal and minimal, and pitch and roll daily buoy records. Both angles were below ±22 deg (*maximum*). The median of the minima and the median of the maxima yielded [−13, +10] deg in pitch and [−12, +11] deg in roll, which are representative of roughly ± 13 deg (± 0.23-rad) angular excursion. Considering first-order Taylor’s approximation, cos(x)≃1 and sin(x)≃x, x=θ, ϕ in Equation (10), this angular excursion yields cos(0.17)=0.97≃1 and sin(0.23)≃0.22, which are 2.5% and 0.8% errors, respectively. This enables us to propose the small-angles approximation, applied to Equation (10), which leads to

(13) $$\hat{z}≃\left[\begin{array}{c} -\theta \\ \phi \\ 1 \end{array}\right].$$

We then define $\vec{r_{zd}}$ as the vector difference between $\hat{d}$ and $\hat{z}$ (see Figure 5),

(14) $$\vec{r_{zd}}=\hat{d}-\hat{z}≃\left[\begin{array}{c} \theta \\ -\phi \\ 0 \end{array}\right]=\theta \left[\begin{array}{c} 1\\ 0\\ 0 \end{array}\right]+\phi \left[\begin{array}{c} 0\\ -1\\ 0 \end{array}\right]=\vec{r_{\theta }}+\vec{r_{\phi }}.$$

Therefore, $\vec{r_{zd}}$ can be expressed as the sum of the two linearly independent vectors $\vec{r_{\theta }}=\theta \left[1,0,0\right]$ and $\vec{r_{\phi }}=\phi \left[0,-1,0\right]$ with modules

(15) $$\left|\vec{r_{\theta }}\right|=\theta ,\left|\vec{r_{\phi }}\right|=\phi .$$

This is shown on an NE plane in Figure 5b.

Moreover, if α→0, sinα≃α and

(16) $$\alpha ≃\sin\left(\alpha \right)=sin\left(\frac{\left|\vec{r_{dz}}\right|}{\left|\hat{z}\right|}\right)=\sin\left(\left|\vec{r_{dz}}\right|\right)≃\left|\vec{r_{dz}}\right|.$$

Because we use unitary vector $\hat{z}$, Equation (15) means that the sought-after eigenangle α is directly a modulus of difference vector $r_{zd}$. Combining Equations (15) and (16), we obtain

(17) $$\alpha ≃\sqrt{\phi^{2}+\theta^{2}}.$$

Equation (17) states that the eigenangle angle can be interpreted as the 1-DoF equivalent formulation for the real 2 DoF problem posed by motion in the pitch and roll angles. This equivalent formulation is in accordance with previous published work [26] in which pitch and roll angular periods were shown experimentally to be correlated (ρ=0.54 in that case).

The PSD of the eigenangle random process, α, is given by a linear combination of pitch and roll PSDs and pitch-to-roll cross-PSD (see Appendix A for details):

(18) $$S_{\alpha ,\alpha }\left(f\right)=S_{\theta ,\theta }\left(f\right)+S_{\phi ,\phi }\left(f\right)-2Im\left[S_{\theta ,\phi }\left(f\right)\right].$$

Because roll(t) and pitch(t) were real-valued time series, $R_{\phi ,\theta }$ is also real-valued and the associated cross-spectral density $S_{\theta ,\phi }$ is Hermitian, i.e., its complex conjugate is equal to the original function with the variable *f* changed in sign, $S_{\theta ,\phi }\left(-f\right)=S_{\theta ,\phi }^{*}\left(f\right)$ or, equivalently, the real part of $S_{\theta ,\phi }\left(f\right)$ is an even function and the imaginary part is an odd function. The latter is important to understand which PSD terms contribute “power” to the random process α (power is computed in units of ${rad}^{2}$, which is not actual physical power but the squared value of signal α).

If we integrate both terms of Equation (18) from *f*=−∞ to *∞*, $\int_{-\infty }^{\infty }S_{\alpha ,\alpha }df$ average power (in units of $rad^{2}$) is given by

(19) $$\sigma_{\alpha }^{2}=\sigma_{\theta }^{2}+\sigma_{\phi }^{2}.$$

Because $Im(S_{\theta ,\phi }\left(f\right))$ is an odd function, integral $\int_{-\infty }^{\infty }Im\left(S_{\theta ,\phi }\right)df$ vanishes out and it emerges that the cross-spectral density does not contribute power to eigenangle random process α; only roll and pitch PSDs do.

### 2.4. PSD Estimation

In order to estimate the PSD of random process α, the Blackman–Tukey method was chosen on account of its computation simplicity and best trade-off between noise rejection and spectral-resolution characteristics [40]. The Blackman–Tukey method $S_{xx}^{BT}\left(f\right)$ consists of smoothing periodogram $P_{xx}\left(f\right)$, here computed through the FFT algorithm, by its convolution with a smoothing window W(f) (rectangular window in this study). It can be formulated as

(20) $$S_{\alpha ,\alpha }^{BT}\left(f\right)=\int_{-1/2}^{1/2}P_{\alpha ,\alpha }\left(\beta \right)W\left(f-\beta \right)d\beta ,$$

with

(21) $$P_{\alpha ,\alpha }\left(f\right)=\frac{1}{L}{\left|\sum_{n=0}^{L-1}\alpha \left(n\right)e^{-j2\pi fn}\right|}^{2}=\frac{1}{L}{\left|FFT(\alpha (n))\right|}^{2},$$

where *L* is the number of FFT samples and “*n*” is shorthand notation for nT, with *T* as the sampling period (T≃0.125 s).

From Equation (18), Blackman–Tukey estimation of the PSD is written as

(22) $$S_{\alpha ,\alpha }^{BT}=S_{\phi ,\phi }^{BT}+S_{\theta ,\theta }^{BT}-2Im\left[S_{\theta ,\phi }^{BT}\right].$$

**Figure 7:**
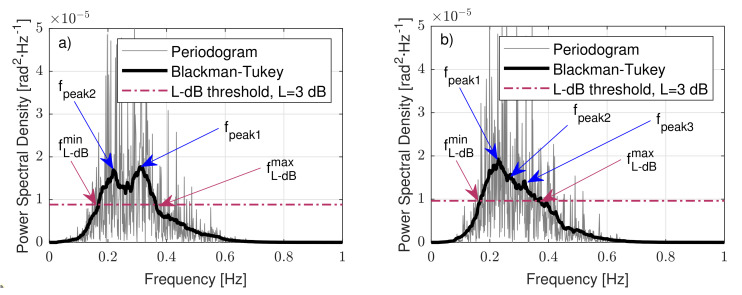
Two examples of power-spectral-density (PSD) estimation by periodogram and Blackman–Tukey method of measured tilt data: (**a**) bimodal case and (**b**) multimodal case. The L-dB threshold and cutoff frequencies, $f_{L-dB}^{min}$ and $f_{L-dB}^{max}$, are also indicated by magenta dashed lines and arrows. L = 3 dB.

Figure 7 shows PSD estimations of two 10 min tilt temporal series of eigenangle α computed by using periodogram ($P_{\alpha \alpha }\left(f\right)$, grey) and the Blackman–Tukey method ($S_{\alpha ,\alpha }^{BT}\left(f\right)$, black).

The IMU sampling frequency was 8 Hz, although some jitter showed up. Roll (ϕ) and pitch (θ) were resampled at a fixed sampling frequency of 10 Hz to ensure a uniform sampling rate and a sampling period that were submultiples of 1 s for convenience. Then, the PSD of α was computed following the Blackman–Tukey estimation method (Equations (20) and (22)).

Figure 7a shows bimodal behaviour for the PSD of α, with two dominant or modal frequencies at 0.22 Hz ($f_{peak2}$) and 0.31 Hz ($f_{peak1}$), equivalently, $T_{2}=1/0.22=4.5$ s and $T_{1}=1/0.30=3.2$ s, respectively, while Figure 7b shows nearly unimodal behaviour with a PSD peak at 0.22 Hz ($f_{peak1}$). The L-dB threshold is also plotted, which was computed as the relative level L-dB below the maximal peak level given by $f_{peak1}$. $f_{L-dB}^{min}$ and $f_{L-dB}^{max}$ denote the minimal and maximal frequency components of the PSD content that were higher than the L-dB threshold (see Section 2.5 for details).

### 2.5. PSD Significant-Wave-Period Estimation

We define the PSD significant wave period as the period associated to the buoy’s eigenangle (Equation (17)) and estimated it by imposing a threshold level on its PSD (Section 2.4). This threshold is found in Section 3 in relation to different well-accepted wave-period oceanographic definitions. Next, we formulated the PSD significant wave-period concept.

Figure 7 shows the buoy’s eigenangle PSD for two different motional cases. In both, it emerged that there was not a single relevant spectral component but multiple ones (labelled $f_{peak,i}$, blue arrows). In order to consider all relevant spectral components contributing power to the significant wave period, we defined an L-dB threshold (quantity “L” to be found) as the relative level L-dB below the maximal peak level of the PSD. This L-dB threshold (L = 3 dB in Figure 7) defines a frequency span, [$f_{L-dB}^{min}$,$f_{L-dB}^{max}$], in which the PSD content is higher than a power factor of $10^{-L/10}$ compared to the peak level. The L-dB method computes the wave period as the average wavelength in the L-dB region. We defined the average wavelength as the arithmetic mean between the maximal and minimal wavelengths:

(23) $$\lambda_{L-dB}=\frac{\lambda_{L-dB}^{max}+\lambda_{L-dB}^{min}}{2}.$$

Introducing the concept of the phase velocity of a wave ($v_{p}$), which is the rate at which the wave propagates in the medium, and that any given phase of the wave (for example, the crest) appears to travel at the phase velocity [41], we can write $\lambda =v_{p}T$, where *T* is the wave period. By inserting this relation into Equation (23) and by using T=1/f, Equation (23) can be rewritten as

(24) $$T_{L-dB}=\frac{\frac{1}{f_{L-dB}^{min}}+\frac{1}{f_{L-dB}^{max}}}{2},$$

which gives the sought-after significant wave-period estimated from the $f_{min}$ and $f_{max}$, L-dB cutoff frequencies of the PSD. Equation (24) can also be interpreted as the harmonic mean of the maximal and minimal cutoff frequencies, $f_{L-dB}^{max}$ and $f_{L-dB}^{min}$, respectively, of the L-dB region (Figure 7).

## 3. Results and Discussion

In order to validate the proposed methodology, $T_{L-dB}$ estimations (Equation (24)) were carried out over tilt (eigenangle) experimental data measured during the whole IJmuiden campaign (80 days) and then compared against reference wave periods measured by the Triaxys^TM^ buoy. Because the Triaxys^TM^ buoy yielded multiple estimations of the wave period according to the different oceanographic definitions (Section 2), we first needed to assess which of these best matched the PSD wave period estimated by using the L-dB method ($T_{L-dB}$, Equation (24)).

To carry out this comparison, three statistical indicators were used: (i) correlation coefficient (ρ), (ii) root-mean-square error (RMSE), and (iii) mean deviation (MD). In the context of WE, a typical sampling period is 10 min; thus, $T_{L-dB}$ was estimated every 10 min. When comparing the significant wave period estimated via the L-dB method and Triaxys^TM^, $T_{L-dB}$ was resampled to the temporal resolution of Triaxys^TM^ (1 h). Root-mean-square error is defined as

(25) $$RMSE=\sqrt{\frac{\sum_{i}{\left(T_{L-dB}-T_{z}\right)}^{2}}{N}}$$

and the mean deviation is defined as

(26) $$MD=\frac{\sum_{i}\left(T_{L-dB}-T_{z}\right)}{N}.$$

**Figure 8:**
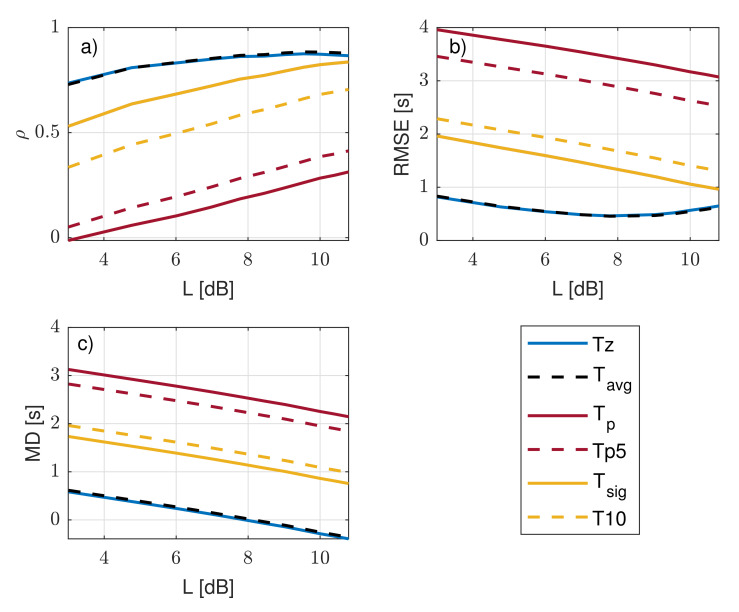
Comparison with 3 statistical indicators of agreement between estimated $T_{L-dB}$ and reference wave periods from IJmuiden campaign’s experimental data at different *L* values: (**a**) correlation coefficient, ρ, as a function of threshold level, L; (**b**) that for root-mean-square error (RMSE) (Equation (25)); and (**c**) that for mean deviation (MD) (Equation (26)).

$T_{L-dB}$ was estimated at L−dB values ranging from 3 to 11 dB and compared against oceanographic wave-period definitions $T_{z}$, $T_{avg}$, $T_{p}$, T10, Te, Tp5, and $T_{sig}$ defined in Section 2 and measured by Triaxys^TM^, which were used as references. Figure 8 shows statistical indicators when comparing $T_{L-dB}$ as a function of L with each of these Triaxys^TM^ reference periods. Figure 8 shows the results of these comparisons in terms of ρ (Figure 8a), RMSE (Figure 8b), and MD (Figure 8c). The zero-crossing and the average-period methods ($T_{z}$ and $T_{avg}$, respectively) from the experiment yielded identical statistical indicators, which is evidenced by the overlapping blue and dashed black lines in the three subfigures (Figure 8a–c). When comparing $T_{z}$ and $T_{avg}$ to $T_{L-dB}$, maximal ρ, minimal RMSE, and MD closest to 0 were evidenced. The largest differences occurred for the wave energy spectrum peak methods ($T_{p5}$ and $T_{p}$). A possible explanation for that is that wave energy spectrum peak methods measured the period corresponding to the peak spectral component and did not consider wave multimodality. Lastly, $T_{10}$ and $T_{sig}$, which consider the highest tenth and third of the wave energy spectrum as the relevant wave spectral components, respectively, showed better agreement than the latter set did ($T_{p5}$ and $T_{p}$), with $T_{sig}$ showing better indicators. $T_{sig}$ showed higher ρ, lower RMSE, and MD closer to 0 than $T_{10}$ due to the broader frequency span. It emerged that the L-dB method best matched $T_{z}$ and $T_{avg}$ (with virtually identical indicators). In the following, the L-dB method is compared with reference to $T_{z}$.

Optimal threshold L was found heuristically using a parametric approach: $T_{L-dB}$ estimations were computed as a function of threshold L spanning from L = 3 to 10 dB for the whole measurement campaign and were then compared statistically against $T_{z}$. Statistically, indicators relating both methods for each threshold value L were computed for each week and each month of the 80 day campaign and, lastly, for the whole campaign. Then, weekly and monthly sets were averaged over all weeks and months, respectively, to yield monthly and weekly ensemble averages (in what follows, the word “ensemble” is skipped). The chosen statistically indicators were the ones above (ρ, RMSE, and MD) along with the slope and intercept point of the linear regression (LR, y = mx + n) between $y=T_{L-dB}$ and $x=T_{z}$.

**Figure 9:**
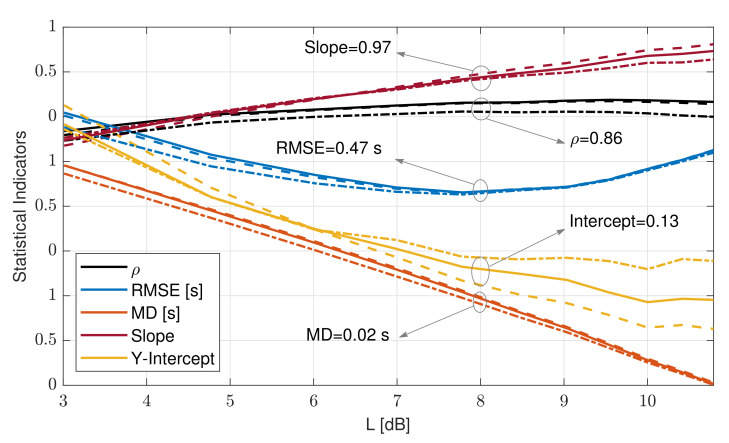
Statistical indicators comparing the L-dB method $T_{L-dB}$ and zero-crossing method $T_{z}$ as a function of threshold value L (dB) parameterized by averaging time (IJmuiden campaign, 29 March–17 June 1920 records). The dashed dots indicate weekly averaged indicators. The dashed line indicates monthly average. The solid trace indicates the indicators computed for the whole 80 day campaign.

Figure 9 shows the statistical indicators computed for the 80 day campaign as weekly and monthly averages and for the whole campaign as a function of threshold L. ρ, RMSE, and LR slope showed similar values for the two time averages considered and for the whole campaign. ρ grew from 0.7 at L = 3 dB to a maximum of 0.86 at 8 dB and onwards. On the other hand, RMSE showed parabolic behavior with minimal RMSE = 0.46 s around L = 7.75 dB. The LR slope showed a linearly increasing trend, with the ideal value of 1 reached at L = 8.5 dB. The LR intercept reached 0 (ideality value) at L = 8.5 dB (weekly averages) and L = 9.5 dB (campaign), although the LR intercept became less relevant because slope deviations from unity (ideal value) are always associated with nonzero intercepts in the regression procedure. Lastly, the MD showed decreasing linear trend and cut the 0 dB baseline at L = 8 dB. Therefore, by choosing threshold L = 8 dB, virtually all ideal indicators’ values were achieved: ρ = 0.86, RMSE = 0.47 s, LR slope = 0.97, and MD ≃ 0.02.

**Figure 10:**
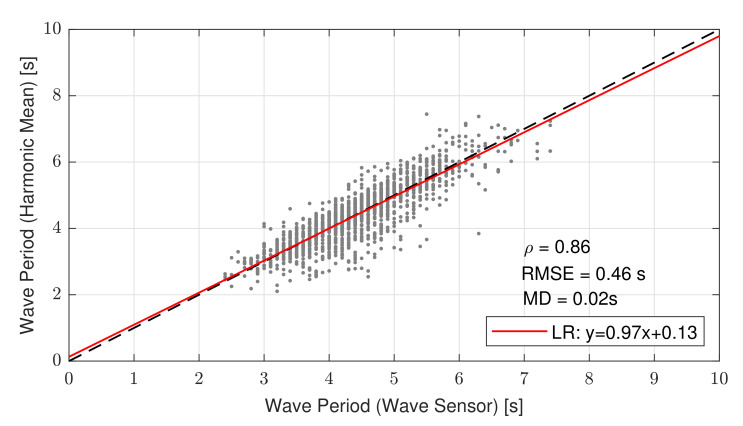
Scatter plot comparing wave period estimated by 8 dB method $T_{8-dB}$ in reference to zero-crossing method $T_{z}$. The red trace shows the linear regression modelling relationship between both methods; the dashed black line is the 1:1 ideal line.

Figure 10 shows the scatter plot between the PSD L-dB method with L = 8 dB, 8-dB method $T_{8-dB}$, and zero-crossing method $T_{z}$. With the chosen 8 dB threshold, both methods reconciled, as evidenced by the indicator values above. Overall, narrowly scattered points represent $T_{8-dB}$ points that did not fall out of the ideal 1:1 line by more than the RMSE value. The straight-line fit had a slope equal to 0.97 and intercept equal to 0.13, all of which yielded virtual coincidence with the ideal 1:1 line.

Despite the good agreement between both methods, which reconciled PSD 8 dB method $T_{8-dB}$ to oceanographic zero-crossing method $T_{z}$, teh scatter-plot outliers accounting for an RMSE approximately equal to 10% of the mean wave period warrants some comments. First, Triaxys^TM^ computation of reference period $T_{z}$ was affected by the buoy’s translational and rotational movements; in our modelling, (Section 2.3 and Section 2.5) only roll and pitch were considered (2 DoF). Second, our methodology was experimentally tested under the assumption of small angles, the median of maximal tilt excursion was ±13 deg (Figure 6), which incurred 2.5% and 0.8% errors when using first-order cosine and sine approximation, respectively. Lastly, the DWL and Triaxys^TM^ reference buoys were 200 m apart during the campaign, which may also have accounted for small wind, current, and wave differences.

## 4. Summary and Conclusions

A new method (L-dB) to estimate the wave period using a spectral analysis of pitch and roll time records measured on a DWL buoy was shown in the context of IJmuiden’s campaign and in comparison to classical oceanographic wave-period estimation methods. This 2 DoF approach assumes quasistatic yaw rotation compared to the wave period and negligible translational motion (see, e.g., Figure 2 mooring scheme).

The 2 DoF buoy motion model enabled formulation of the so-called eigenangle, which is the buoy tilt angle around the eigenaxis of rotation of the lidar buoy (Euler’s rotation theorem). Specifically, the eigenangle is the angle between the down (D) component of the north–east–down fixed coordinate system (IMU frame of reference) and the Z component (downwards) of the buoy’s XYZ moving coordinate system.

Under the practical approximation of small angles, the eigenangle can be computed as the quadratic sum of pitch and roll angles (Equation (17)), and it can be modelled as a complex-number random process, hence assimilating into its real and imaginary components both pitch and roll time series. Histograms records of daily maximal and daily minimal pitch and roll angles (160 records) yielded angular excursions of [−22, +22] deg (min/max values) and [−13, +11] deg (median values), with these values showing quantitative description of the small-angle approximation used in our study.

Under these conditions, the PSD of the eigenangle $S_{\alpha ,\alpha }$ was derived (see Appendix A as the linear combination of pitch and roll PSDs ($S_{\theta ,\theta }$ and $S_{\phi ,\phi }$, respectively) and the pitch-to-roll cross-PSD ($S_{\theta ,\phi }$). PSD was computed by applying the Blackman–Tukey method over 10 min data segments, and wave period $T_{L-dB}$ was computed from the $f_{min}$ and $f_{max}$ cutoff frequencies of the PSD (Equation (24)) at which the spectral components dropped off L decibels from the peak level.

The proposed L-dB method, which is rooted in the spectral context, was compared to different wave-period definitions from the oceanographic context, namely, the mean zero-crossing, average, and peak periods, among others. The study was carried out by using threshold L as parameterization, and the correlation coefficient, ρ, RMSE, MD, and LR slope, and intercept as statistical indicators. The L-dB method was indistinctly in close agreement with the zero-crossing and average wave-period definitions, and a threshold value L = 8 dB exhibited the best indicators when comparing the L-dB and zero-crossing methods over daily, weekly, and whole-campaign averages, hence reconciling the spectral approach to the oceanographic one.

Lastly, when comparing our 8 dB method ($T_{8-dB}$) with the zero-crossing one ($T_{z}$), the LR slope was 0.97 and the intercept was 0.13, which virtually matched the ideal 1:1 line (Figure 10). Regarding the statistical indicators above, the 8 dB method yielded fairly good results with ρ = 0.86, RMSE = 0.46 s (compared to a mean wave-period over the campaign of 4.39 s), and MD = 0.02 s. In spite of this low RMSE value, a few outliers departed up to 2 s from the ideal line. We speculate that his misestimation was due to the assumption of 2 DoF (pitch and roll) in our methodology in comparison to the 3 DoF (heave, roll, and pitch) used by Triaxys (and in the literature) to compute the wave period, the small-angle approximation, and the 200 m distance between the DWL and Triaxys buoy references. All in all, the proposed L-dB method allows floating wind lidars to provide increased knowledge on the sea state (i.e., sea period), which can enhance wind measurements and reduce offshore wind farms deployment cost. Such knowledge could be assimilated into offshore wind measurements and can be used to complement mesoscale wind prediction models, which could help improve ship safety under strong wind conditions. Particularly, the IJmuiden sea lock has recently been the object of a study in order to explore techniques to improve the safety of ships mooring and navigating nearby [42], which could benefit from these offshore wind lidar measurements.

## Data Availability

The data presented in this study are available on request from the corresponding author, roca@tsc.upc.edu.

## Abbreviations

The following abbreviations are used in this manuscript:

DOAJ	Directory of open access journals
DoF	Degree of freedom
DWL	Doppler wind lidar
FEM	Fourier expansion method
HWS	Horizontal Wind Speed
LR	Linear regression
MD	Mean deviation
MEM	Maximum entropy method
NED	North–east–down
FFT	Fast Fourier transform
IMU	Inertial measurement unit
MDPI	Multidisciplinary Digital Publishing Institute
Metmast	Meteorological mast
PSD	Power spectral density
RMSE	Root-mean-square error
WE	Wind Energy

## Appendix A. Power-Spectral-Density Derivation

Next, we compute the PSD associated to the time variations of eigenangle α, given by Equation (17). Using the superposition principle described by Equation (17) and illustrated in Figure 5a, where pitch and roll contributions add up in quadrature, α can be described in a straightforward form by means of time-varying complex function:

(A1) α(t)=θ(t)−jϕ(t),

where bold is used to denote a complex number.

The PSD of a complex random process, α(t), is defined as the Fourier transform of the autocorrelation function. Formally,

(A2) $$S_{\alpha ,\alpha }\left(f\right)=\int_{-\infty }^{\infty }R_{\alpha ,\alpha }\left(\tau \right)e^{-i2\pi f\tau }d\tau ,$$

where $R_{\alpha ,\alpha }\left(\tau \right)$ is the autocorrelation of α [40], τ is the time lag, and *f* is the frequency. The autocorrelation of a complex process is defined as [40]

(A3) $$R_{x,x}\left(\tau \right)=E\left[x\left(t\right)x^{*}\left(t+\tau \right)\right],$$

where * denotes the complex conjugate.

By inserting Equation (A1) into Equation (A3) above, the autocorrelation function of random complex process α(t) yields

(A4) $$R_{\alpha ,\alpha }\left(\tau \right)=R_{\phi ,\phi }\left(\tau \right)+R_{\theta ,\theta }\left(\tau \right)+j\left[R_{\theta ,\phi }\left(\tau \right)-R_{\phi ,\theta }\left(\tau \right)\right].$$

Using $R_{\phi ,\theta }\left(\tau \right)=R_{\theta ,\phi }^{*}\left(-\tau \right)$ (using a similar definition to Equation (A3) but for the cross-correlation between random processes θ(t) and ϕ(t) [43]), Equation (A4) above reduces to

(A5) $$R_{\alpha ,\alpha }\left(\tau \right)=R_{\phi ,\phi }\left(\tau \right)+R_{\theta ,\theta }\left(\tau \right)+j\left[R_{\theta ,\phi }\left(\tau \right)-R_{\phi ,\theta }^{*}\left(-\tau \right)\right].$$

By inserting Equation (A5) into the PSD definition of Equation (A2) and using

(i) the cross-PSD (also called cross spectral density) between two processes x(t) and y(t) is the Fourier transform (FT) of the cross-correlation function, $S_{x,y}=\int_{-\infty }^{\infty }R_{x,y}\left(\tau \right)e^{-i2\pi f\tau }d\tau$, and
(ii) $R_{\theta ,\phi }^{*}\left(\tau \right)\to S_{\theta ,\phi }^{*}\left(f\right)$ according to the FT conjugation property, $x^{*}\left(t\right)\to X^{*}\left(-f\right)$, with *X* the FT of signal x(t) and the arrow symbol denoting FT,

the PSD of α is obtained as

(A6) $$S_{\alpha ,\alpha }\left(f\right)=S_{\theta ,\theta }\left(f\right)+S_{\phi ,\phi }\left(f\right)+j\left[S_{\theta ,\phi }\left(f\right)-S_{\theta ,\phi }^{*}\left(f\right)\right].$$

The terms into square brackets on the right side of Equation (A6) can be recognized as the complex subtraction, $z-z^{*}=2iIm\left(z\right)$, with $z=S_{\theta ,\phi }\left(f\right)$ and $Im(\dot{)}$ denoting the imaginary part. Therefore,

(A7) $$S_{\alpha ,\alpha }\left(f\right)=S_{\theta ,\theta }\left(f\right)+S_{\phi ,\phi }\left(f\right)-2Im\left[S_{\theta ,\phi }\left(f\right)\right],$$

with units of [$rad^{2}/Hz$].
